# Assessment of knowledge and quality of essential newborn care practices in La Dade Kotopon Municipality, Ghana

**DOI:** 10.1371/journal.pone.0237820

**Published:** 2020-08-25

**Authors:** John Ayete-Nyampong, Emilia Asuquo Udofia

**Affiliations:** 1 School of Public Health, University of Ghana, Legon, Accra, Ghana; 2 Department of Community Health, University of Ghana Medical School, College of Health Sciences, University of Ghana, Accra, Ghana; University of Michigan Medical School, UNITED STATES

## Abstract

Majority of neonatal deaths in developing countries have been associated with inappropriate or poorly practiced newborn care, specifically safe cord care, optimal thermal care and early initiation and practice of exclusive breastfeeding. There is limited information about the quality of these essential newborn care practices in Accra, Ghana. The main objective of this study was to assess the knowledge about and quality of essential newborn care practices (ENC) and determine related factors in La Dade Kotopon Municipal Assembly, Accra, Ghana. A questionnaire-based, cross-sectional study was conducted among 423 mothers and caregivers in two hospitals to assess safe cord care, optimal thermal care and exclusive breastfeeding. Knowledge was assessed using eight statements regarding ENC and categorized as ‘Adequate knowledge’ and ‘Inadequate knowledge’ using a composite score. Practices were similarly categorized as ‘Good’ and ‘Poor’ ENC. Data were exported from Microsoft Excel into STATA version 15 for statistical analysis. Descriptive statistics were generated and inferential analysis was done using chi-square test and logistic regression to determine factors associated with good ENC at 95% confidence level. All respondents sampled participated in the study. A total of 263 (62%) respondents had adequate knowledge and 308 (73%) respondents practiced appropriate newborn care (‘Good’ ENC). The likelihood of appropriate newborn care practices was lower among mothers/caregivers who were unemployed (AOR = 0.13, 95% CI: 0.09–0.26), who had a home delivery (AOR = 0.17, 95% CI: 0.11–0.69) and made their first antenatal visit in the third trimester (AOR = 0.02, 95% CI: 0.01–0.35) compared to their counterparts. Knowledge of ENC was not associated with practice in this study. Appropriate newborn care practices were relatively high among the respondents. Improving sources of livelihood and targeted education to encourage early antenatal visits and facility-based births might improve newborn care where it is inadequate.

## Introduction

The first 28 days of life is an important period for child survival and accounts for about 50–70% of infant mortality [1]. In 2016, nearly 2.6 million deaths occurred, accounting for 46% of all under-five deaths globally [2]. A greater percentage of the neonatal deaths take place between the first and seventh day of life, with almost a million deaths occurring on the first day and a similar number of deaths within the next six days [3]. The major causes of neonatal deaths globally include infections (36%), pre-term births (28%) and birth asphyxia (23%) [4].

The World Health Organization (WHO) recommends that women should endeavour to deliver and receive postnatal care (PNC) in a healthcare facility within the first 24 hours [5]. In the case of home deliveries, mothers should receive a home visit within 24 hours of delivery. A minimum of three extra postnatal contacts are recommended for all mothers and newborns on the third day (48–72 hours), between the first and second week after birth (7^th^ -14^th^ day), and six weeks after birth [5]. These recommendations have been made to ensure positive health outcomes for the mother and the baby as well as consistent utilization of key newborn care practices with the aim of reducing neonatal mortality.

Essential newborn care (ENC) practices include hygienic cord care, optimal thermal care and early initiation of breastfeeding [6]. Over 30% of neonatal deaths can be prevented through the uptake and utilization of these key practices according to the Lancet Neonatal Health Series [7]. WHO recommendations state that dry cord care (safe cord care) is practiced when nothing is applied on the cord stump of the newborn [8]. The umbilical cord when not fully healed is a major avenue for infections mainly caused by bacteria from the maternal genital tract or from unhygienic and unsafe care practices [9]. Traditional cord-care practices which involve the application of relatively unsafe substances can increase the likelihood for infections in the newborns [9]. On the other hand, dry umbilical cord care for newborns can effectively reduce neonatal mortality resulting from cord related infections [2].

Optimal thermal care is required because the thermoregulatory mechanisms of babies are underdeveloped [10], hence they lack the ability to regulate their own body temperature without thermal protection, with preterm babies being at high risk [11]. It involves delaying bathing a newborn for 24 hours, skin to skin contact, drying with a clean towel and wrapping the baby appropriately. Early bathing has been associated with increased risk of hypothermia in the newborn [12]. Skin-to-skin contact between mother and child is very crucial because it keeps the baby warm thereby preventing hypothermia, keeps the baby calm, improves feeding outcomes and strengthens the bond between the mother and her baby [13].

Early initiation of breast-feeding ensures that the child is protected against a wide range of pathogens prior to acquisition of active immunity from vaccination [14]. Early and exclusive breastfeeding (EBF) is crucial for the survival of infants as it also promotes healthy development of the brain, improves cognitive and mental performance and promotes physical development. According to WHO recommendations, breastfeeding should be initiated within an hour after delivery and should be sustained exclusively for 6 months. Thereafter, complementary feeds should be given to meet evolving nutritional needs while continuing breastfeeding up to two years and beyond [13]. Late initiation of breastfeeding is associated with an increased risk of neonatal mortality of 33%, which can be prevented by early and exclusive breastfeeding [15, 16].

It is therefore necessary that these newborn care practices are undertaken in the health facilities and at home and that they are safe and acceptable. The quality of these newborn care practices must also meet globally recommended standards.

The WHO has defined quality of care as cited in [17]:

“*The extent to which health care services provided to individuals and patient populations improve desired health outcomes*. *In order to achieve this*, *health care needs to be safe*, *effective*, *timely*, *efficient*, *equitable*, *and people-centered*.”

Apart from training and equipping healthcare providers, the knowledge of mothers and caregivers regarding essential newborn care is critical to the quality of essential newborn care. Health education provided during the antenatal and postnatal period improves the knowledge of postnatal mother regarding essential newborn care practices [18]. This will ensure that during the most critical period of the child’s life, safe, acceptable and essential care practices that will ensure their survival are practiced properly and consistently. Therefore, the present study sought to assess the quality of essential newborn care practices in La Dade Kotopon Municipality in the Greater Accra Region, Ghana.

## Methods

### Ethics approval and consent to participate

Ethical approval for this study was sought and granted by the Ghana Health Service Ethical Review Board (GHS-ERC 060/04/19). Permission was sought from facility authorities and granted before undertaking the study. Participants were informed about the objectives and methods in the study and a written informed consent was obtained from the participating mothers and caregivers prior to participation. Participants were informed about their right to withdraw from the study without any repercussions.

### Study design

A descriptive, cross sectional design was employed in this study. Interviews were conducted with structured questionnaires. The reason for employing the descriptive design was to provide accurate description of the newborn care practices of mothers and caregivers through analysis and interpretation of responses to a questionnaire-based survey of postnatal attendees. The design also offered convenience in terms of cost and time for conduct of the study.

### Study setting

The La Dade Kotopon Municipal Assembly (LADMA) has its administrative capital at La and has a population of 183,528 as of 2010 [19]. Two hospitals in the municipality were selected based on the high average monthly attendance namely La General Hospital (LGH) and Police Hospital (PH). These study sites were chosen purposively based on the high monthly average of postnatal attendants. According to the District Health Information Management System (DHIMS), the average monthly postnatal attendances at PH and at LGH were 237 and 382 respectively in 2018.

### Study population

Mothers and caregivers with babies aged six weeks or less who visited the postnatal clinics at the two health facilities from 7^th^ June to 10^th^ July, 2019.

### Sample size estimation

The sample size was obtained by applying a formula for cross-sectional studies [20]. A prevalence of 50% was assumed for any of the newborn care practices [21], at 95% confidence level with a margin of error of 5% and non-response rate of 10% based on a similar study [22]. The calculated sample size was 423. This sample size was distributed between the two hospitals proportional to their average postnatal monthly attendances.

### Sampling technique

Consecutive sampling was used to select respondents at both hospitals. Each eligible postnatal clinical attendee was selected until the required sample size for each hospital was attained. Participants due for postnatal sessions were determined using postnatal registers. Midwives in-charge working at the postnatal units of both facilities assisted to eliminate duplications from repeat visits.

### Data collection

An interviewer-administered, structured questionnaire was used for data collection. This questionnaire was adapted from literature [23, 24] and modified to align with the study objectives and WHO Essential Newborn Care Guidelines. Two research assistants were recruited and trained to assist with the data collection in addition to the clinic nurses. English language was used for communication and local dialects (Ga, Ewe and Twi or Fante) were used when necessary. The questionnaire was pre-tested on twenty postnatal mothers at the University of Ghana Hospital, Legon and modifications required were made before the commencement of data collection (see S1 File).

### Quality control

Questionnaires were thoroughly examined at the close of each data collection session to ascertain consistency and completeness. Periodic checks were conducted on the research assistants during interviews to ensure that they maintained good ethical conduct. The mothers were provided with identification numbers to prevent a repeat interview. The nurses kept a list and used it to cross-check with the register on subsequent days to prevent duplication.

### Data processing and analysis

Data was entered into Microsoft Excel and exported into STATA version 15 for statistical analysis. Each essential newborn care practice i.e. early and exclusive breastfeeding, thermal care and umbilical cord care was assessed using a series of close ended questions. Each appropriate response scored one (1) point and an inappropriate response scored zero (0) point. Good practice for early and exclusive breastfeeding referred to early initiation of breastfeeding within an hour of birth and given on demand for the first six months of life. No other fluids or solids were given excluding oral rehydration solution, multivitamin drops or syrups, minerals or medicines. Good practice for thermal care referred to immediate drying and wrapping of the newborn, with skin to skin contact following birth and postponing the bathing until after 24 hours. Good practice for cord care referred to keeping the cord dry and not applying any substance to the cord stump. All good practices were based on WHO recommendations [5, 14]. Mothers who gave correct responses to at least 75% of questions were deemed as having a ‘Good practice’, otherwise it was adjudged ‘Poor practice’. This method of scoring and categorization was based on a similar study by [24].

A composite score was generated for the knowledge about essential newborn care practices (ENCP), used to categorize the variable into ‘Adequate’ and ‘Inadequate’ knowledge. Independent variables included mother’s age (<24 years, 25–29 years, 30–34 years, 35–49 years), level of education (≤Junior High School (JHS)/Middle School (MS), Senior High School (SHS)/O’Level/A’ Level, Tertiary), occupation (Employed, Self-employed, Not employed), place of delivery (Home/Hospital), number of ANC visits (≤4/≥5) and number of postnatal clinic visits (1, 2, ≥3). Descriptive statistics were presented as frequencies and percentages. Chi-square analysis was used to determine the relationship between the outcome variable (essential newborn care practices) and independent variables (mothers age, education, antenatal care and postnatal care visits attended), followed by logistic regression analysis to control for potential confounders. Conclusions were drawn based on the adjusted Odd’s ratios and 95% confidence limits constructed around the estimates.

## Results

### Socio-demographic characteristics

A total of 423 mothers and caregivers participated with 100% response rate. The mean (s.d.) age of respondents was 28.01±6.11 (Table 1). Two hundred and forty-one respondents were married (57%) and the predominant religion was Christianity, 354(83.70%). Majority of the respondents, 188 (44.40%) and 148 (48.10%) partners had achieved secondary education and had one child, 181 (42.80%). The respondents were predominantly Ga-speaking, 181 (42.80%) and mainly resided in La, 142 (33.60%). One hundred and ninety-seven respondents were self-employed 197 (46.60%), with most of them being traders, caterers and seamstresses (Table 1).

**Table 1:** Socio-demographic characteristics of respondents.

		Good newborn care practices		Poor newborn care practices		Total		Chi-square statistic, p-value
Characteristics		Frequency	Percent	Frequency	Percent	Frequency	Percent	
	**Age (years)**					m(s.d.) = 28.01±6.11		χ2 = 10.13, p = 0.03
	≤ 24	89	73.40	34	26.60	123	22.20	
	25–29	89	76.72	27	23.28	116	27.40	
	30–34	64	62.14	39	37.86	103	24.30	
	35–49	66	81.48	15	18.52	81	19.10	
	**Marital Status**							χ2 = 1.73, p = 0.63
	Single	85	73.91	30	26.09	115	27.20	
	Married	178	73.86	63	26.14	241	57.00	
	Co-habiting	41	66.13	21	33.87	62	14.70	
	Widowed	4	80.00	1	20.00	5	1.20	
	**Number of Children**							χ2 = 3.38, p = 0.34
	1	125	69.06	56	30.94	181	42.80	
	2	101	75.37	33	24.63	134	31.70	
	3	51	79.69	13	20.31	64	15.10	
	4+	31	70.45	13	29.55	44	10.40	
	**Religion**							χ2 = 5.17, p = 0.07
	Muslim	40	57.97	29	42.03	69	16.30	
	Christian	268	75.70	86	24.30	354	83.70	
	**Ethnicity**							χ2 = 2.73, p = 0.54
	Ga	122	67.40	59	32.60	181	42.80	
	Akan	115	82.73	24	17.27	139	32.90	
	Ewe	41	69.49	18	30.51	59	13.90	
	Others	30	68.18	14	31.82	44	10.40	
	**Education (Respondent)**							χ2 = 9.05, p = 0.04
	≤JHS/MS	48	28.41	121	71.59	169	39.89	
	SHS/O/A-level	54	28.72	134	71.28	188	44.40	
	Tertiary	53	80.30	13	19.70	66	15.60	
	**Education (Partner)**							χ2 = 12.76, p = 0.01
	≤JHS/MS	18	48.65	19	51.35	37	11.90	
	SHS/O/A-level	114	76.00	36	24.00	150	48.10	
	Tertiary	96	76.80	29	23.20	125	40.10	
	**Occupation**							χ2 = 8.40, p = 0.02
	Employed	118	78.67	32	21.33	150	35.50	
	Self-employed	144	73.10	53	26.90	197	46.60	
	Unemployed	46	60.53	30	39.47	76	18.00	
	**Residence**							χ2 = 6.26, p = 0.08
	La	114	80.28	28	19.72	142	33.60	
	Teshie	77	76.23	24	23.77	101	23.90	
	Nungua	31	53.44	27	46.56	58	13.70	
	Trade fair	24	68.57	11	31.43	35	8.30	
	Other	62	71.26	25	28.74	87	20.60	

m(s.d.) = mean (standard deviation), JHS = junior high school, MS = middle school, SHS = senior high school, O/A-level = ordinary level/advanced level.

### Maternal health services

Majority of the respondents delivered in a hospital 406 (95.98%) and achieved five or more antenatal visits, 224 (54.63%). Antenatal care attendance in the first trimester was reported by 218 (53.30%) respondents (Table 2). In all, one hundred and sixty-one (38.10%) respondents achieved two postnatal visits for the index child and majority also made postnatal visits for the older child, 218 (51.50%). The ages of the babies ranged from 3 days to 6weeks, most in the range of 5–9 days (Table 2).

**Table 2:** Antenatal and postnatal attendance of respondents.

Antenatal care	Frequency	Percent
**Place of delivery**		
Home	17	4.02
Hospital	406	95.98
**Antenatal attendance**		
Yes	410	97.00
No	13	3.00
**Number of antenatal visits**		
≤4	186	45.37
≥5	224	54.63
**Trimester of first antenatal visit**		
1^st^ trimester	218	53.30
2^nd^ trimester	163	39.70
3^rd^ trimester	29	7.00
**Postnatal care**		
**Age of baby (days)**		
1–7	196	46.34
8–14	106	25.06
15–21	54	12.76
> 21	67	15.84
**No. of postnatal visits**		
1	116	27.40
2	161	38.10
≥3	146	34.50
**Postnatal attendance for older child**		
Yes	218	51.50
No	205	48.50

### Essential newborn care practices

#### Breastfeeding

Overall, 368 (87%) respondents practiced exclusive breastfeeding. Initiation of breastfeeding within an hour after delivery was reported by 267 (63.10%) respondents. Three hundred and ninety-one (92%) respondents reported they had fed their babies with colostrum, and 220 (52%) respondents breastfed their babies based on their discretion or when necessary. Hygienic practices undertaken by the respondents prior to breastfeeding included bathing at least once a day 84(19.90%), washing hands 210(49.60%), and cleaning the breast with a cloth or towel 104(24.60%). Overall, the number of respondents who reported appropriate breastfeeding practices was 308 (72.81%).

#### Cord care

Three hundred and eighty-seven (91.50%) respondents claimed that the cord was cut with a pair of scissors, with 82 (19.40%) of them unaware of whether the item was clean or not. The use of a cord clamp on the cord was reported by 412 (97.40%) respondents, with 353 (83.50%) respondents claiming the clamp was clean before use. Majority of respondents, 71 (88.75%), used methylated spirit for dressing the cord. While the application of chlorhexidine gel is a preferred practice in areas with high neonatal mortality (30 or more neonatal deaths per 1000 live births) or if cultural norms require application of a substance on the stump [5], 39.50% of respondents reported using it and a few others (2.30%) applied substances such as mud, mustard oil and saliva. Only six percent of respondents adhered to the WHO recommendations of dry cord care by not applying anything to the cord. Overall, 279 (66%) respondents reported practicing good cord care.

#### Thermal care

Majority of the respondents, 265(62.60%) affirmed that the first bath of their baby took place after 24 hours of birth. Forty-nine (11.60%) respondents reported that the first bath took place immediately after the birth of the baby. Almost all the respondents, 421 (99.50%) claimed that the baby was dried and wrapped after birth. Some practices used in keeping the baby warm included keeping baby close to mother’s skin, 79 (18.70%), wrapping baby with warm clothes, 311(73.50%), and bathing baby with warm water, 33 (7.80%). Overall, the number of respondents who practiced good thermal care was 416 (98.35%).

### Quality of newborn care practices

The three essential newborn care practices (breastfeeding exclusively, cord and thermal care) of respondents were categorized into ‘Good’ and ‘Poor’ newborn care practices using a composite score. Overall, a total of 308(72.80%) respondents practiced good newborn care (Table 3).

**Table 3:** Quality of newborn care practices.

Practices	Good	Poor
	n (%)	n (%)
Breastfeeding	306 (72.30)	117 (27.70)
Cord care	279 (65.90)	144 (34.10)
Thermal care	416 (98.40)	7 (1.60)
Essential newborn care	308 (72.80)	115 (27.20)

### Factors associated with newborn care practices

Age, education level, education level of partner, occupation, place of delivery, previous postnatal attendance, number of antenatal visits, trimester of first antenatal visit and number of postnatal visits were associated with essential newborn care practices using the chi-square test. After regression analysis, four variables significantly influenced essential newborn care practices. Respondents who were unemployed (AOR = 0.13, 95% CI: 0.09–0.26), who had a home delivery (AOR = 0.17, 95% CI: 0.11–0.69) and made their first antenatal visit in the third trimester (AOR = 0.02, 95% CI: 0.01–0.35) were less likely than their counterparts to demonstrate good newborn care practices (Table 4). Knowledge of ENC was not associated with the practice of ENC in bivariate analysis (χ2 = 3.67, p = 0.055) and therefore it was not included in the regression model.

**Table 4:** Logistic regression analysis of selected variables and essential newborn care practices.

Variables	Essential newborn care practices (n = 423)		COR (95% CI)	AOR (95% CI)
	Poor: n (%)	Good: n (%)		
**Age (years)**				
≤24 (ref)	34 (27.65)	89 (72.35)	1	1
25–29	27 (23.28)	89 (76.72)	1.29 (0.72–1.68)	0.36 (0.22–1.43)
30–34	39 (37.86)	64 (62.14)	0.63 (0.19–1.02)	**0.52 (0.51–1.24)**
35–49	15 (18.52)	66 (81.48)	1.63 (0.48–2.33)	----------
**Education level**				
Senior High/O-level/A-level (ref)	54 (28.72)	134 (71.28)	1	1
≤Junior High/ middle school	48 (28.40)	121 (71.59)	0.97 (0.45–1.56)	0.54 (0.31–2.82)
Tertiary	13 (19.70)	53 (80.30)	1.04 (0.16–1.41)	1.31 (0.79–2.72)
**Education level of partner**				
Senior High/O-level/A-level (ref)	36 (24.00)	114 (76.00)	1	1
≤Junior High/middle school	19 (51.36)	18 (48.64)	0.51 (0.38–4.26)	0.33 (0.19–5.16)
Tertiary	29 (23.20)	96 (73.08)	4.27 (1.37–4.22)	3.99 (1.28–3.44)*
**Occupation**				
Self-employed (ref)	53 (26.90)	144 (73.10)	1	1
Employed	32 (21.33)	118 (78.67)	1.92 (0.37–1.55)	0.77 (0.25–1.67)
Unemployed	30 (39.47)	46 (60.53)	0.37 (0.12–0.65)	0.13 (0.09–0.26)*
**Place of delivery**				
Hospital (ref)	104 (25.62)	302 (74.38)	1	1
Home	11 (64.71)	6 (35.29)	0.14 (0.11–0.39)**	0.17 (0.11–0.69)*
**Antenatal Attendance**				
Yes (ref)	107 (25.91)	306 (74.09)	1	
No	8 (80.00)	2 (20.00)	0.08 (0.02–0.42)*	----
**Antenatal visits**				
5 and above (ref)	44 (19.47)	182 (80.53)	1	1
4	23 (24.73)	70 (75.27)	0.82 (0.56–2.71)*	0.76 (0.34–3.61)
≤3	40 (42.56)	54 (57.44)	0.29 (0.08–1.61)	0.13 (0.09–1.48)
**Trimester of first antenatal visit**				
1^st^ trimester (ref)	51 (23.18)	169 (76.82)	1	1
2^nd^ trimester	41 (25.00)	123 (75.00)	0.91 (0.56–1.45)	1.13 (0.49–2.57)
3^rd^ trimester	15 (51.72)	14 (48.28)	0.28 (0.13–0.62)*	0.02 (0.01–0.35)*
**Postnatal visits**				
2 (ref)	36 (22.36)	125 (77.64)	1	1
≥3	49 (33.56)	97 (66.44)	0.27 (0.26–0.43)*	1.34 (0.35–2.64)
1	30 (25.86)	86 (74.14)	0.63 (0.09–1.72)	0.59 (0.12–2.53)

Dependent variable: Essential newborn practices, criterion level: p< 0.05, (*p<0.05, **p<0.001).

Ref = reference variable.

### Knowledge about essential newborn care practices

To test their knowledge of essential newborn care practices, mothers responded to eight statements regarding safe and acceptable practices for newborns with three options ‘Agreed’, ‘Disagree’ and ‘Don’t know’. A composite score was used to categorize respondents as having ‘Adequate knowledge’ or ‘Inadequate knowledge’. Overall, 263 (62.20%) respondents had adequate knowledge about newborn care practices (Table 5).

**Table 5:** Knowledge of mothers and caregivers about essential newborn care practices.

Statement	Agree		Disagree		Don't know	
	N	%	N	%	N	%
First breastfeed should be between 30 minutes and an hour after delivery	366	86.50	27	6.40	30	7.10
Newborns should be fed with first yellowish milk (colostrum)	387	91.50	15	3.50	21	5.00
Newborns should be exclusively breastfed from birth to six months	344	81.30	67	15.80	12	2.80
The cord of your baby must always be dressed and covered	117	27.70	235	55.60	71	16.80
Bleeding, discharges, redness and swelling in your baby's cord is normal	57	13.50	344	81.30	22	5.20
The umbilical cord must be exposed to air and kept dry at all times	234	55.30	105	24.80	84	19.90
The baby's first bath must be delayed at least 24 hours after birth	195	46.10	134	31.70	94	22.20
The baby must always be wrapped after birth and kept in close contact to the mother’s skin	421	99.50	2	0.50	0	0.00

### Factors associated with knowledge about essential newborn care practices

Bivariate analysis indicated statistically significant relationships with marital status, education level, education level of partner, occupation, previous postnatal attendance, number of antenatal visits, number of postnatal visits and previous postnatal attendance (Table 6). Three variables demonstrated independent effects in the logistic regression model: single status (AOR = 0.34, 95%CI: 0.19–0.59), first antenatal visit in the 3^rd^ trimester (AOR = 0.08, 95%CI: 0.01–0.69) and making one postnatal visit (AOR = 0.34, 95%CI: 0.22–0.43) (Table 6). Women with these characteristics were less likely to have adequate knowledge about essential newborn care.

**Table 6:** Logistic regression showing association between selected variables and knowledge about ENC.

	Knowledge about essential newborn care practices (n = 423)		COR (95% CI)	AOR (95% CI)
	Inadequate: n (%)	Adequate: n (%)		
**Marital status**				
Married (ref)	73 (30.29)	168 (69.71)	1	1
Single	53 (46.09)	62 (53.91)	0.25 (0.13–2.67)	0.34 (0.19–0.59)*
Co-habiting	34 (54.84)	28 (45.16)	1.45 (0.71–3.26)	0.92 (0.63–4.82)
**Level of education**				
Senior High/O-level/A-level	78 (41.49)	110 (58.51)	1	1
≤Junior High/ middle school	64 (37.87)	105 (62.13)	0.63 (0.48–1.59)	1.02 (0.54–3.77)
Tertiary	18 (27.27)	48 (72.73)	-----	1.34 (1.07–4.91)*
**Level of education (partner)**				
Senior High/O-level/A-level	52 (34.67)	98 (65.33)	1	1
≤Junior High/middle school	23 (62.16)	14 (37.84)	1.33 (0.66–2.80)	0.89 (0.52–4.56)
Tertiary	39 (31.20)	86 (68.80)	1.75 (1.22–3.42)	1.58 (0.90–2.17)
**Occupation**				
Self-employed (ref)	68 (34.52)	129 (65.48)	1	1
Employed	47 (31.33)	103 (68.67)	1.11 (0.63–1.28)	0.92 (0.31–1.68)
Unemployed	45 (59.21)	31 (40.79)	0.44 (0.26–0.79)*	0.32 (0.08–1.52)
**Antenatal Attendance**				
**Yes (ref)**	151 (36.56)	262 (63.44)	1	1
**No**	9 (90.00)	1 (10.00)	0.64 (0.03–0.51)*	----
**Antenatal visits**				
5 and above (ref)	88 (38.94)	138 (61.06)	1	1
4	43 (46.24)	50 (53.76)	0.21 (0.27–2.36)	0.41 (0.33–3.11)
≤3	20 (15.22)	74 (84.78)	0.82 (0.46–1.37)	0.61 (0.19–1.06)
**Trimester of first antenatal visit**				
1^st^ trimester (ref)	87 (39.55)	133 (60.45)	1	1
2^nd^ trimester	50 (30.49)	114 (69.51)	1.49 (0.97–2.29)	1.99 (0.96–4.16)
3^rd^ trimester	14 (48.28)	15 (51.72)	0.70 (0.32–1.52)	0.08 (0.01–0.69)*
**Postnatal visits**				
**2** (ref)	48 (29.81)	113 (70.19)	1	1
1	62 (53.45)	54 (46.55)	**0.42 (0.06–0.82)**	0.34 (0.22–0.43)*
≥**3**	50 (40.95)	96 (59.05)	3.76 (1.15–4.24)	2.91 (0.79–3.68)
**Postnatal for older child**				
**Yes (ref)**	64 (29.36)	154 (70.64)	1	1
**No**	96 (46.83)	109 (53.17)	0.47 (0.32–0.70)**	0.37 (0.35–1.68)

Dependent variable: Knowledge about ENC, criterion level: p< 0.05, (*p<0.05, **p<0.001)

Ref = reference variable.

## Discussion

The present study aimed to assess the quality of newborn practices being employed by mothers and care givers in the La Dade Kotopon Municipality. Overall, the practice of essential newborn was relatively high, 72.80% compared to lower rates reported in a study in Mandura district, Northwest Ethiopia (40.60%) and in an earlier study conducted in Lawra district, Ghana (15.80%) [25]. This might have been due to the fact that the present study took place in an urban setting compared to the rural areas, where prevalence of good newborn care practices was reportedly lower. The practice of essential newborn care was higher (81.10%) in a study undertaken in Mekelle City, Northern Ethiopia [24].

Majority of respondents in the present study practiced exclusive breastfeeding similar to an earlier study in Madina (87% vs. 75%) [26]. A study conducted in India by [27] reported that 92.78% of respondents exclusively breastfed their babies. The World Health Organization (WHO) recommends that babies are exclusively breast fed for at least six months, especially in low resource settings, which was prevalent in the present study. Counter-intuitively [28] reported that 44% of Egyptian newborns received supplemental substances as pre-lacteal feeds, particularly during the first seven days of life. In a cross-sectional study conducted in Nepal, 80.40% of newborns were given pre-lacteal feeds within the first 14 days of life and 63.10% of the respondents started breastfeeding within an hour after delivery [29]. In this study, colostrum was given by majority of the respondents, with others opting to give plain water, gripe water, formula feed and sugar water instead. This was similar to the study by [27], where all the respondents reported giving their babies colostrum. WHO universally recommends that colostrum, the first milk that flows within the first few days after delivery is the perfect food for every newborn. A study in Southern Ethiopia revealed that colostrum was not given and mostly discarded by more than half of the respondents [30]. Other studies show similar trends with majority of respondents discarding the colostrum and instead feeding the baby with glucose water, water, formula feeds, castor oil etc [31, 32].

According to [13], clean, dry cord care is recommended for all newborns in health facilities and at home in low neonatal mortality settings (30 neonatal deaths per 1000 live births). The neonatal mortality rate in Ghana is 29 neonatal deaths per 1000 live births, which qualifies it as a low neonatal mortality setting [19]. Only six percent of respondents adhered to the WHO recommendations of dry cord care by not applying anything to the cord. Methylated spirit has been widely used and accepted for cord care in Ghana, therefore it is not surprising that 52% of respondents applied methylated spirit for cord care. Although the application of chlorhexidine gel is permitted for cord care of newborn delivered at home in settings with high neonatal mortality, 39.50% of respondents reported using it. A few others (2.30%) applied substances such as mud, mustard oil and saliva. a study conducted in Lawra district, it was reported that majority of mothers applied various things like shea-butter, shea-butter mixed with powder to the cord stump [25]. Reasons provided for this practice include hastening the healing process, preventing cord stump from emitting odious smells and preventing infections.

Another WHO recommendation concerning cord care is the use of a sterilized instrument in cutting the cord and a clean instrument in tying the cord. The use of clean instruments in cutting and tying the cord was commonly practiced, 78.5% and 83.5% respectively. This can be attributed to the fact that majority of the births took place in a health facility and as such processes such as cutting and tying the cord were mostly handled by the midwives. Some studies show similar trends where majority of the instruments used in cutting and tying the cord were sterilized [25, 30].

WHO recommendations for optimal thermal care include delayed bathing for the baby for up to 24 hours (or at least 6 hours for cultural reasons), drying and wrapping the baby as well skin to skin contact in the first hour of life [13]. The study showed that majority of the babies (62.60%) had their first bath 24–48 hours after birth. Similar studies in Ethiopia showed high rates of delayed bathing [24, 33]. In a study undertaken in Pakistan, 86% of respondents reported that their babies were given their first bath within 24 hours of birth [28]. Similarly, a study conducted by [34] reported that 58% of newborns had early baths. Early bathing was associated with beliefs that the baby is ‘dirty’ due to the vernix, that it is necessary to eradicate body odor in later life and it is necessary in shaping the baby’s head in an earlier study in Brong Ahafo region, Ghana [35]. Nearly all the respondents reported that their babies were dried and wrapped after birth. The methods employed in keeping the baby warm included keeping the baby close to the mothers’ skin (skin-to-skin contact), wrapping the baby with warm clothes and bathing the baby with warm water. WHO recommends skin-to-skin contact between mother and child early after delivery [13]. As was the case in the present study and in similar research undertaken in Tanzania, only a few mothers practiced skin-to-skin contact [27]. In a previous study, respondents demonstrated knowledge about the necessity of optimal thermal care but majority of the babies born at home were not dried and wrapped promptly. Additionally, most of them were bathed early after delivery (93%) and only a few were placed skin-to-skin (10%) [35].

### Factors associated with essential newborn care practices

Many studies have shown that factors such as age of the mother at birth, mother’s level of education, as well as facility deliveries strongly influence the quality of newborn care practiced [25, 36, 37]. Although age and level of education did not significantly influence essential newborn care practice in the present study, respondents who had a home birth were less likely to practice essential newborn care practices compared to those who had a facility delivery [AOR = 0.17, 95%CI: 0.11–0.69]. Other studies conducted have reported similar findings [36, 38]. In Ghana currently, 43% of all births take place at home [39]. This implies that inappropriate newborn care practices could remain a problem in this sub-set of births, placing them at a potential risk of neonatal morbidity and mortality.

Those who were unemployed were less likely to practice good newborn care [AOR = 0.13, 95%CI: 0.09–0.26] compared to those who were self-employed. A study in Uganda supports this finding, where women of higher socioeconomic status were more likely to access quality health services and consequently practiced good newborn care [38]. Delayed commencement of antenatal care was associated with a lower likelihood of practicing essential newborn care [AOR = 0.02, 95%CI: 0.01–0.35]. Another study in Northern Ghana indicated a similar pattern, where women who attended ANC late were less likely to practice good newborn care compared to those who initiated antenatal care early [39].

### Knowledge of essential newborn care practices

Knowledge about ENC was adjudged adequate for 62.20% of respondents for all essential newborn care practices assessed. A similar study conducted in the Bawku municipality of the Upper East region of Ghana reported that 70.50% of respondents demonstrated good knowledge of ENC practices [22]. In another study conducted in India, 91.50% of postnatal women had adequate knowledge regarding neonatal care practices, which was higher than that reported in the present study [40]. Marital status was significantly associated with adequate knowledge of essential newborn care in the final regression model [AOR = 0.34, 95%CI: 0.19–0.59]. Single women less likely to have good knowledge about ENC compared to married women. This finding supports a study conducted in Ethiopia by [41] which showed that married women were more likely to have more information and hence better knowledge about safe and ENC practices as compared to those who were not married.

Late commencement of antenatal care influenced the knowledge of ENC negatively. Respondents who started antenatal care in the third trimester were less likely to have adequate knowledge compared to those who started in the first trimester (AOR = 0.08, 95%CI: 0.01–0.69). This was probably due to these attendees missing out on important information about ENC which is often discussed early on during antenatal sessions. Women who reported making at least one postnatal visit [AOR = 0.34, 95%CI: 0.22–0.43] were less likely to have adequate knowledge compared to women who made one postnatal visit. Studies have found that antenatal and postnatal clinic attendance are significantly associated with better knowledge and practice of ENC [24, 26]. This might further suggest that, in spite of the desirability of educating mothers about essential newborn care throughout the maternity care spectrum, the second and fourth postnatal visits may represent important windows of intervention.

Some of the gaps identified from the newborn care practices assessed were: mixed breastfeeding, bathing the baby immediately after birth and poor hygienic practices undertaken before handling the cord. In relation to the knowledge regarding essential newborn care practices, deficient areas were delaying the first bath of the baby for up to 24 hours and keeping the cord dry and uncovered. To address these shortcomings, home visits can be made by community health nurses to identify mothers who were unable to attend antenatal and postnatal clinics, and to serve as a means of encouraging them to make visits as well as promote health facility-based deliveries. Certain crucial messages regarding newborn care should be repeated during each antenatal and postnatal clinic day so mothers who are unable to attend all sessions can benefit from the repeated messages. Additionally, qualitative research should be conducted in the area of newborn care to adequately assess the influence of other factors not examined here such as culture.

Limitations in the present study include the use of consecutive sampling which precludes external generalization of the study findings. The results were based on self-report and may not be as objective as actual observation of the practices. Logistic constraints made the use of observations difficult as the women would have to be followed to their residence and they lived in different areas within the district. Finally, the study did not explore the influence of culture, in which case a qualitative approach might have been desirable. This could be a subject for future research.

## Conclusion

The study findings indicated adequate knowledge and good practice of early and exclusive breastfeeding, safe cord care and optimal thermal care were in accordance with WHO Essential Newborn Care guidelines for majority of respondents. Factors associated with essential newborn care practices were occupation, marital status, place of delivery and trimester of first antenatal visit. Knowledge about essential newborn was inadequate if women were single, had their first antenatal visit in the third trimester and had a single postnatal visit. Improving sources of livelihood and targeted education to encourage early antenatal visits and facility-based births might improve newborn care where it is inadequate.

## Supporting information

S1 File Questionnaire for the study (24KB). (DOCX)

## Abbreviations

ANC: Antenatal care
DHIMS: District Health Information Management System
LADMA: La Dade Kotopon Municipal Assembly
PNC: Postnatal care
WHO: World Health Organization
