# Supplementary material for: Assessment of knowledge and quality of essential newborn care practices in La Dade Kotopon Municipality, Ghana
Source: PLoS One. 2020 Aug 25;15(8):e0237820. doi: 10.1371/journal.pone.0237820 (PMC7454570; doi:10.1371/journal.pone.0237820)
Supplement: S1 File — (DOCX) [file pone.0237820.s001.docx]

**Participants Consent Form**

**STUDY TITLE:**

Assessment of the Quality of Newborn Care Practices in the La Dade Kotopon Municipality.

**PARTICIPANT’S STATEMENT**

I acknowledge that I have read or have had the purpose and contents of the participants’ Information Sheet read and satisfactorily explained to me in a language I understand (English
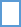
/Ga
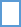
/Twi
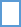
). I fully understand the contents and any potential implications as well as my right to change my mind (thus, withdraw from the study) even after I have signed/thumb printed this form.

I voluntarily agree to be part of this research.

Respondent Name/Initials…………………………………………..

Signature/thumbprint/Mark………………………………. Date…………………………….

**INTERPRETER’S STATEMENT**

The purpose and content of the participants’ Information Sheet has been explained to the afore named participant to the best of my ability in (English
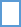
/Ga
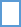
/Twi
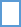
). All questions and clarifications sought by the participant and answers were also duly interpreted to his/her satisfaction .

Name of Interviewer …………………………………..

Signature of Interviewer ……………………………….

Date……………………………………………………..

**STATEMENT OF WITNESS**

I was present when the purpose and contents of the Participants’ Information Sheet was read and explained satisfactorily to the participant in (English
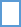
/Ga
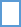
/Twi
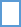
).

I confirmed that she was given the opportunity to ask questions/seek clarifications and same were duly answered to her satisfaction before voluntarily agreeing to be part of the research.

Name…………………………………………..

Signature/thumbprint/Mark………………………………. Date…………………………….

**INVESTIGATOR STATEMENT AND SIGNATURE**

I certify that, the details of this study at large have been thoroughly explained to the participant and all questions and clarifications raised were duly attended to.

Researcher Name……………………………………

Signature…………………………………………… Date…………………….

**Questionnaire**

**SCHOOL OF PUBLIC HEALTH**

**COLLEGE OF HEALTH SCIENCES**

**UNIVERSITY OF GHANA**

**PROJECT TOPIC:**

**ASSESSMENT OF THE QUALITY OF NEWBORN CARE PRACTICES IN LA DADE KOTOPON MUNICIPAL DISTRICT**

*Dear Respondent,*

*I am John Ayete-Nyampong, a student of the University of Ghana and I would need your co-operation to answer this questionnaire on “****Assessment of the quality of newborn care practices in La Dade Kotopon municipal district.”*** *Be assured that information provided would be used strictly for academic purposes and confidentiality is fully assured. The questionnaire should take about 15 minutes to fill*

Questionnaire number: _______ Interview Date: ___/___/_2019

**SECTION A: SOCIO- DEMOGRAPHIC DATA**

1. Age of respondent (at last birthday)……………………
2. Marital Status

a) Single [ ] b) Married [ ] c) Co-Habiting [ ] d) Divorced [ ] e) Widowed [ ]

1. Number of children
2. 1 [ ] b) 2 [ ] c) 3 [ ] d) >3 [ ]
3. Religion
4. Muslim [ ] b) Christian [ ] c) Traditional [ ] d) Others (specify)…...
5. Tribe
6. Ga [ ] b) Akan [ ] c) Ewe [ ] d) Others (specify)…
7. Your educational level

a) None [ ] b) Primary [ ] c) Junior high/middle school [ ] d) Senior High/O-level/A-level [ ] e) Tertiary [ ]

1. Education level of husband

a) None [ ] b) Primary [ ] c) Junior high/middle school [ ] d) Senior High/O-level/A-level [ ] e) Tertiary [ ]

1. Occupation
2. Employed [ ] b) self-Employed [ ] c) Unemployed [ ]
3. Place of residence………………

**SECTION B: ANTENATAL AND POSTNATAL ATTENDANCE**

1. Place of delivery
2. Hospital [ ] b) Home [ ] c) Others (specify) ………………….
3. Did you attend antenatal care for this baby?
4. Yes [ ] b) No [ ]
5. How many visits?
6. One [ ] b) Two [ ] c) Three [ ] d) Four [ ] e) Five [ ]
7. Which trimester did you start antenatal clinic
8. 1^st^ trimester [ ] b) 2^nd^ trimester [ ] c) 3^rd^ trimester [ ]
9. I) How old is your baby?...................

II) Number of postnatal visits:

1. One [ ] b) Two [ ] c) Three [ ] d) Four [ ] e) Five [ ]

III) Compliance with required postnatal visits( For interviewer based on responses in (i) and (ii) above)

1. Compliant [ ] b) Non-compliant [ ]
2. Any previous postnatal attendance for an older child?
3. Yes [ ] b) No [ ]

**SECTION C : ESSENTIAL NEWBORN CARE PRACTICES**

1. (I) What type of breastfeeding do you practice
2. Exclusive [ ] b) Mixed [ ]

(II) If you practice mixed feeding, what is the main reason?

1. Insufficient breast milk [ ] b) Pain in nipple [ ] c) Job demands [ ] d) Others (specify)…………..
2. How early after delivery did you initiate breastfeeding
3. Within an hour [ ] b) After an hour [ ] c) After two hours [ ] d) Three hours and above [ ]
4. If breast-feeding was initiated an hour or more after delivery what was the reason?
5. Fatigue [ ] b) Complication after delivery [ ] c) Birth attendant’s advice [ ] d) Others (specify)…………………..
6. Was the baby fed with the first yellowish breast milk (colostrum)?
7. Yes [ ] b) No [ ] c) Don’t know [ ]
8. How often do you breastfeed your baby?
9. Only when baby cries [ ] b) After every three hours [ ] c) After every four hours [ ] d) Others (specify) ………………….
10. What hygienic practices do you undertake before breastfeeding?
11. Bath at least once a day [ ] b) Apply lotions or oils on breast [ ] c) Wash hands [ ] d) Clean breast with cloth or towel [ ]
12. Is the baby burped after each feed
13. Yes [ ] b) No [ ]
14. (I) Is the baby given any other fluid besides breast milk?
15. Yes [ ] b) No [ ]

(II) If yes what fluid is given to the baby

1. Plain water [ ] b) Gripe water [ ] c) Sugar water [ ] d) Other (specify)……………….
2. How can you tell if breast feeding is going well
3. Baby looks healthy [ ] b) Baby is calm and relaxed [ ] c) Baby gains weight [ ] d) Baby restless or crying [ ]
4. What was used to cut the cord after delivery
5. Scissors [ ] b) Blade [ ] c) Knife [ ] d) Other (specify) ……………..
6. Was the above mentioned material clean?
7. Yes [ ] b) No [ ] c) Don’t know [ ]
8. What was used to tie the cord after birth
9. String [ ] b) Rubber band [ ] c) Cord clamp [ ] d) Wire [ ] d) Other (specify) …………
10. Was the above mentioned material clean
11. Yes [ ] b) No [ ] c) Don’t know [ ]
12. What main substance/ medication do you use in dressing your baby’s cord?
13. Methylated spirit [ ] b) Mud [ ] c) Saliva [ ] d) Mustard oil [ ] e) Cow dung [ ]

f) Other (specify)………………..

1. Do you wash your hands before caring for the baby’s cord
2. Always [ ] b) Sometimes [ ] c) Scarcely [ ] d) Not at all [ ]
3. When did you give the baby its first bath
4. Immediately after birth [ ] b) 6 hours after birth [ ] c) 7-24 hours after birth [ ] d) 48 hours after birth [ ] e) Other (specify) ……………..
5. Was the baby wrapped after birth
6. Yes [ ] b) No [ ] c) Don’t know [ ]
7. How do you keep your newborn warm?
8. Keep baby close to mothers’ skin [ ] b) Wrap baby with warm clothes [ ] c) Bath baby with warm water [ ] d) Other (specify) ………………..

**SECTION D(KNOWLEDGE ON ESSENTIAL NEWBORN CARE)**

1. First breastfeed should be between 30minutes and an hour after delivery
2. Agree [ ] b) Disagree [ ] c) Don’t know [ ]
3. Newborns should be fed with first yellowish milk(colostrum)
4. Agree [ ] b) Disagree [ ] c) Don’t know [ ]
5. Newborns should be exclusively breastfed from birth to six months
6. Agree [ ] b) Disagree [ ] c) Don’t know [ ]
7. The cord of your baby must always be dressed and covered
8. Agree [ ] b) Disagree [ ] c) Don’t know [ ]
9. Bleeding, discharges, redness and swelling in your baby’s cord is normal
10. Agree [ ] b) Disagree [ ] c) Don’t know [ ]
11. The umbilical cord must be exposed to air and kept dry at all times
12. Agree [ ] b) Disagree [ ] c) Don’t know [ ]
13. The baby’s first bath must delayed at least 24 hours after birth
14. Agree [ ] b) Disagree [ ] c) Don’t know [ ]
15. The baby must always be wrapped after birth and kept in close contact to the mother’s skin?
16. Agree [ ] b) Disagree [ ] c) Don’t know [ ]
17. Do you have any questions or comments you wish to make?

………………………………………………………………………………

………………………………………………………………………………

………………………………………………………………………………

*Thank you for your participation.*
